# Content validation of the Adapted Cycles Intervention Program (PROCICLOS-A) for children with Speech Sound Disorder – Activities

**DOI:** 10.1590/2317-1782/e20240378en

**Published:** 2026-02-09

**Authors:** Carolina Kuntz Ayub, Haydée Fiszbein Wertzner

**Affiliations:** 1 Programa de Pós-graduação em Ciências da Reabilitação, Departamento de Fonoaudiologia, Fisioterapia e Terapia Ocupacional, Faculdade de Medicina, Universidade de São Paulo – USP - São Paulo (SP), Brasil.; 2 Departamento de Fonoaudiologia, Fisioterapia e Terapia Ocupacional, Faculdade de Medicina, Universidade de São Paulo – USP - São Paulo (SP), Brasil.

**Keywords:** Speech, Language and Hearing Sciences, Speech, Speech Sound Disorder, Speech Therapy, Phonotherapy, Phonological Disorder

## Abstract

**Purpose:**

This document aims to describe and validate the activities of the Adapted Cycles Intervention Program (PROCICLOS-A) for children with speech sound disorders.

**Method:**

The study employs a prospective, cross-sectional design focusing on quantitative analysis. PROCICLOS-A consists of 12 sessions, with a specific phonological process selected as the target for every six sessions. In total, two phonological processes and two target sounds are chosen for each cycle, resulting in four target sounds for the intervention. Each of the 12 sessions includes six types of activities: auditory bombardment, conducted at the beginning and end of each session, training in the production of the target sound, focusing on the articulation zone, mode, and voicing, auditory recognition of the target sound and auditory discrimination using minimal pairs, activities with minimal pairs to aid in understanding the phonological rule, training with words containing the target sound in initial, medial, and final positions, and phonological awareness activities. Specific materials were developed to implement each of these strategies. A total of twenty expert judges (EJs) participated in evaluating the activities, assessing all five activities along with their 14 respective strategies. To analyze the level of agreement among the judges, we utilized an alternate coefficient known as AC1, proposed by Gwet (2014). This analysis focused on the judges' responses related to the activities.

**Results:**

The agreement among the ten EJs for the activities was measured at 0.7125, indicating a moderate level of agreement.

**Conclusion:**

The activities utilized in PROCICLOS-A for children with speech disorders demonstrated a good level of agreement for all the materials produced.

## INTRODUCTION

### Speech sound disorder

Children’s phonological acquisition occurs during their development, when the phonetic inventory of their linguistic system increases mediated by auditory perception, motor production of sounds, and cognitive-linguistic aspects, resulting in the organization of phonological rules^(1)^.

Deletions and substitutions of one or more sounds in the language, called error patterns or phonological processes, may occur during development and are overcome over time. When a child maintains phonological processes beyond the expected age, there is an indication of impairment in their phonological system, a condition characterized as speech sound disorder (SSD), which is common in children, especially preschoolers. SSD is an umbrella term used to refer to any combination of difficulties with auditory perception, motor production, and phonological representations of speech sounds, directly impacting the way the person speaks^(2)^.

SSD is highly prevalent in preschoolers and schoolchildren. Among the various types, the cognitive-linguistic (or phonological) SSD is the most prevalent in this age group^(3)^. Much research has been done on intervention approaches that focus on idiopathic phonological SSD^(4)^.

### Intervention approaches

The literature presents several intervention approaches for idiopathic phonological SSD (phonological SSD). Two important aspects to consider when choosing an intervention approach are its clear objectives and well-described elements, enabling its application, whether in a clinical or research setting^(5)^. A study by Hegarty et al.^(6)^ warns that many speech-language-hearing pathologists may feel insecure about choosing the best intervention approach because they lack knowledge about its effectiveness regarding the intended treatment. Considering that an intervention seeks to reorganize the phonological system of children with phonological SSD, the authors observed that most professionals opt for conventional approaches focusing on minimal pairs, motor production, and phonological awareness.

An intervention approach widely used by speech-language-hearing pathologists, called Cycles^(7)^, aims to facilitate the acquisition of phonological patterns through the careful selection of phonemes in words that would be used in auditory and kinesthetic activities to enhance the child's phonological skills. Each therapy session includes auditory bombardment strategies and varied activities involving words with the target sound. Tactile and auditory cues are provided during the activities, aiming for successful, correct sound production, with their frequency decreasing as the child improves.

### Content validation of the intervention instrument

Research on intervention approaches points to the need to consider evidence-based practice^(8,9)^, for which an important issue is the research results that indicate the effectiveness of an intervention approach. An assessment instrument or intervention approach with evidence of content validity can provide sufficient opportunities to work on the proposed skills^(10)^.

Baker et al.^(5)^ proposed a taxonomy to identify common and uncommon elements in interventions for phonological SSD. The taxonomy aims to identify which elements are described in an intervention approach and the relevance, impact, and purpose of each element for the application of a given approach. To this end, they selected 15 intervention approaches that were analyzed regarding their structures, objectives, and applicability to provide transparent descriptions for both clinicians and researchers. The authors cite the importance of clearly identifying which elements are described in an intervention approach, as well as the relevance, impact, and purpose of each of these elements for the application of a given approach. The elements analyzed may vary across approaches regarding the objective and focus of the intervention, the objectives of the strategies, and the activities chosen, which can influence their effectiveness and use in research and clinical care. They also emphasize that, in the process of developing an intervention approach, it is necessary not only to demonstrate its effectiveness but also to ensure that each part clearly presents its objective and that the activities and strategies achieve their intended purpose. Another study^(9)^ indicates that implementing interventions that include a manual, training, and appropriate materials for their application can increase the use of evidence-based practices among clinical professionals and researchers.

Thus, an important step in assessing the effectiveness and transparency of an intervention program is through its content validation. This consists of evaluating pre-selected items, assessed based on the degree to which each element of an instrument is relevant and representative of the target population. This process determines the accuracy of specific results based on their measurement. Its objective is to determine whether the instrument in question meets all its proposed objectives, using appropriate psychometric procedures^(11)^.

Two aspects must be considered in the validation of any instrument: the reliability and validity of what is being studied. For Phelan and Wren^(12)^, reliability is the degree to which a tool presents stable and consistent results. Among its subtypes, interrater reliability assesses the degree to which different judges (or raters) agree on their choices, being most appropriate for evaluating illustrations, photographs, or other non-textual material prepared for inclusion in a publication. However, according to the same authors, reliability, while an important measure, is not sufficient. The authors further explain that content validation is used to ensure the extent to which the instrument being evaluated measures what it purports to do, and which items are appropriate for that instrument.

Some authors also argue that content validation should encompass three phases: identification of domains, formation of items, and construction of the instrument^(13)^. They also suggest that content validation should be done through evaluation by a committee of judges formed by at least five experts, who should receive specific instructions on how to evaluate each item by completing a questionnaire^(13)^.

By verifying the content validity of the activities and strategies of an intervention approach, this study aims to demonstrate that such an approach can effectively address its intended purpose.

It hypothesizes that the activities and their strategies proposed in PROCICLOS-A are appropriate for stimulating each skill addressed. This study aimed to describe and validate the activities of the Adapted Cycles Intervention Program (PROCICLOS-A) for children with SSD.

## METHODS

This is a prospective, cross-sectional, quantitative study whose materials relate to a specific intervention approach, the Adapted Cycles Intervention Program (PROCICLOS-A). The study was approved by the Ethics Committee CAAE 87068318.2.0000.0065, number 6.500.529. An online consent form was prepared and attached to the form sent to the expert judges (EJ) to be completed before judging the activities and strategies.

### PROCICLOS-A

PROCICLOS-A is an intervention program based on the Hodson and Paden cycles approach^(14)^, focusing on the interaction of cognitive-linguistic, perceptive, and motor speech production processes. The central objective of this type of intervention, which permeates the processes of acquisition and mastery of language sounds and their phonological rules, is to ensure a gradual process. This means that new sounds are introduced for development even if the previous ones have not yet been fully learned. The intervention occurs in a cyclical format: every two sessions, the target sound is changed, with no accuracy criteria required for this change to occur. This type of intervention approach also includes activities such as auditory bombardment, articulatory training, and phonological awareness^(14-18)^.

PROCICLOS-A is a review of the Adapted Cycles Approach intervention proposal^(19)^, which in turn is an adaptation of the cycles approach proposed by Hodson and Paden^(14)^.

PROCICLOS-A was developed in a research laboratory to be applied to children with idiopathic phonological SSD. The program aims to eliminate speech unintelligibility through activities that stimulate auditory perception of sounds and provide the necessary cues for the adequate production of speech sounds using the phonological rules of the language expected for the child's age – i.e., an integrated approach, as suggested by Wren et al^(4)^.

**Figure 1:**
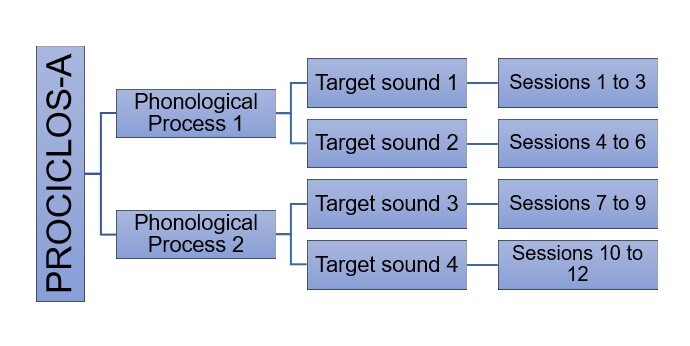
Flowchart of the distribution of phonological processes and target sounds in PROCICLOS-A

As with the cycle approach, PROCICLOS-A adopts a cyclical strategy. In PROCICLOS-A, two phonological processes and two target sounds for each phonological process (totaling four target sounds) are selected to be worked on during the 12 sessions (Figure 1). The process begins with the most frequent process – i.e., the one that is most severely compromising speech intelligibility. Phonological processes that are eliminated earlier in development are usually selected. As for target sounds, stimulable ones are chosen first. PROCICLOS-A works on eliminating phonological processes by using minimal pairs with minimal opposition, with a difference in only one phoneme and a single contrastive feature, such as /vaka/ vs. /faka/ (Portuguese for “cow” vs. “knife”).

Each of the 12 sessions features six types of activities that promote different skills: auditory bombardment (at the beginning and end of the session); placement of the target sound; auditory recognition of the target sound and auditory discrimination with minimal pairs; activities with minimal pairs with minimal opposition for rule comprehension; word practice with the target sound in initial, medial, and final positions; and phonological awareness activities. Chart 1 shows the objectives and strategies of each activity carried out in the 12 PROCICLOS-A sessions.

**Chart 1:** Objective and strategies of the PROCICLOS-A activities

Activity	Objective	Strategy
Activity 1: “Auditory Bombardment”	To have the child listen carefully to words with the target sound.	Reading disyllabic words that begin with the target sounds of the phonological process being worked on.
Activity 2: "Presentation and Articulatory Production of the Target Sound".	To help the child produce sounds, using multimodal facilitating cues – i.e., auditory, visual, and tactile cues.	2.1. Presenting the target sound – A) Cards guiding the production of speech sound.
		2.1. Presenting the target sound – B) Speech ultrasound.
		2.2. Practicing articulatory production of the target sound.
Activity 3: "Target Sound Recognition and Auditory Discrimination with Minimal Pairs".	To help the child recognize and discriminate the target sound in words.	3.1. Auditory Recognition of the Target Sound – A) Jumping Game
		3.1. Auditory Recognition of the Target Sound – B) Right Slap Game
		3.2 Auditory Discrimination of the Target Sound – Grouping Game
Activity 4: "Minimal Pairs Strategy to Understand the Rule".	To help the child understand and use the phonological rule.	4. A) Memory Game
		4. B) Domino
		4. C) Go Fish Game
Activity 5: "Practicing with words with the target sound in initial, medial, and final position".	To work on the correct production of the target sound in initial, medial, and final positions, to stimulate phonological working memory, and to assist in the recognition of the sounds worked on.	5. A) Bingo
		5. B) Riddles
		5. C) Phoneme Trail
Activity 6: "Phonological Awareness".	To reflect on the target sound and its phonological representation.	6. A) Dice
		6. B) Syllabic Segmentation

### Subjects

Twenty speech-language-hearing EJs participated in the content validation process of the PROCICLOS-A intervention program. The inclusion criteria were speech-language-hearing pathologists with a master's and a doctoral degree (or doctoral students), with experience working on phonological SSD. EJs were invited via WhatsApp or email. The researcher initially introduced herself in the email and then briefly explained the study. The message concluded with an invitation to participate as an EJ and provided links to each form. All EJs who agreed to participate signed an informed consent form, presented to each EJ to respond to at the beginning of each form. The form included the question: "Do you agree to participate in this research?", followed by two options: "I agree" or "I disagree."

### Procedures

A specific form was prepared to analyze the activities and strategies and sent via a link to the EJs. Once each EJ had completed the form, the responses were organized into an Excel table.

### Activities

The form completed by each of the 20 EJs contained 14 questions relating to the strategies of each activity.

**Figure 2:**
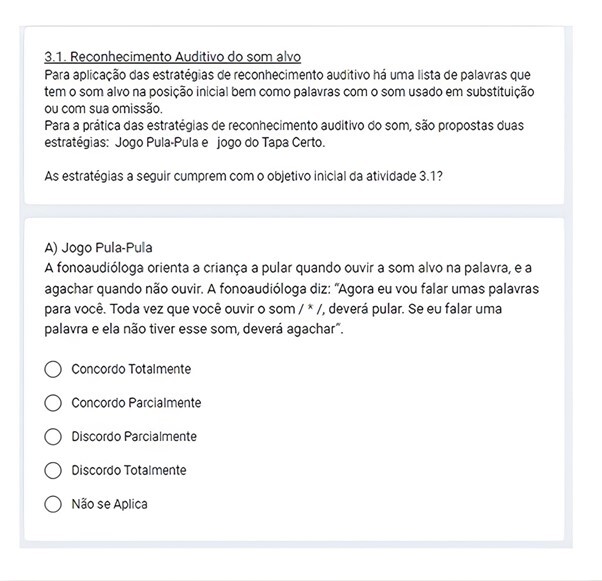
Sample Form for “Activities”

The form included a brief explanation of each activity and the skill(s) each activity was intended to stimulate – the intended objective. Next, the strategies to achieve the activity’s objectives were presented. The EJs had to analyze and decide whether the strategies were appropriate to achieve the objectives proposed in each activity. This analysis was performed using the Likert scale: "I totally agree," "I partially agree," "I partially disagree," "I totally disagree," and "Not applicable." An example of this form is shown in Figure 2.

### Statistical analysis

The agreement between the EJs was calculated using Fleiss' Kappa statistics, which is an extension of Cohen's Kappa statistics for more than two judges. However, according to Gama^(20)^, Santos^(21)^, and Wongpakaran et al.^(22)^, Cohen's and Fleiss' Kappa coefficients perform deficiently in certain situations, such as this study, in which the proportion of occurrence of a response category is very high when compared to the others, resulting in low coefficient values, despite the sum of the proportions in which the EJs agreed being high. To adapt the analyses, Gama^(20)^, Santos^(21)^, and Wongpakaran et al.^(22)^ recommend the use of an alternative coefficient, Gwet’s AC1^(23)^, applied in this study. The classification of agreement according to the AC1 value is presented in Table 1.

**Table 1:** Assessment of agreement according to the value of the AC1 coefficient

Coefficient value	Agreement
< 0.20	Slight
0.21 to 0.40	Fair
0.41 to 0.60	Moderate
0.61 to 0.80	Substantial
0.81 to 1.00	Almost perfect

## RESULTS

Responses were obtained from the 20 EJs for each of the submitted forms. All EJs responded to the "Activities" form. The agreement between EJs is presented considering each analyzed item.

### Description of EJ agreement for the activities

The analysis of the agreement between the 20 EJs for the PROCICLOS-A activities, with their respective strategies, indicated an AC1 value of 0.7125, considered a moderate agreement value. To verify the influence of each activity on the AC1 coefficient value, it was recalculated by excluding one activity at a time. If the AC1 value obtained by excluding an activity was lower than the original value obtained with all activities (AC1 0.7125), the result would indicate that the excluded activity contributed to better overall agreement. However, if the value was greater than 0.7125, it indicated that the excluded activity worsened overall agreement among the judges when maintained. Table 2 shows that with activities 2.1 – A), 2.2, 3.1 – A), 3.1 – B), 4 – A), 4 – B), 4 – C), and 6.2 – B), despite slightly worsening the general agreement index, the AC1 value remains as moderate agreement, indicating that the activities are adequate and, therefore, can be maintained.

**Table 2:** . Value of the AC1 coefficient after excluding each activity

Activities	AC1
2.1 – A)	**0.7193**
2.1 – B)	0.6996
2.2	**0.7292**
3.1 – A)	**0.7247**
3.1 – B)	**0.7193**
3.2 – A)	0.6912
4 – A)	**0.7129**
4 – B)	**0.7193**
4 – C)	**0.7193**
5 – A)	0.6976
5 – B)	0.7057
5 – C)	0.6885
6 – A)	0.7057
6 – B)	**0.7129**

The analysis of the proportion of occurrence of EJ response alternatives indicates that, for all activities, the response “I totally agree” ranged from 70% to 100%. Only two items – 2.1 – B) and 3.2 – A), respectively – had a “I partially disagree” and a “Not applicable” response (Table 3).

**Table 3:** Proportion of occurrence of responses from expert judges

Activities		I totally agree	I partially agree	I partially disagree	Not applicable	Total
2.1	Presenting the target sound – A)	16 (80%)	4 (20%)	0 (0%)	0 (0%)	20 (100%)
	Presenting the target sound – B)	17 (85%)	2 (10%)	1 (5%)	0 (0%)	20 (100%)
2.2	Practicing the articulatory production of the target sound	14 (70%)	6 (30%)	0 (0%)	0 (0%)	20 (100%)
3.1	A) Jumping Game	15 (75%)	5 (25%)	0 (0%)	0 (0%)	20 (100%)
	B) Right Slap Game	16 (80%)	4 (20%)	0 (0%)	0 (0%)	20 (100%)
3.2	A) Grouping Game	18 (90%)	1 (5%)	0 (0%)	1 (5%)	20 (100%)
4	A) Memory Game	17 (85%)	3 (15%)	0 (0%)	0 (0%)	20 (100%)
	B) Domino	16 (80%)	4 (20%)	0 (0%)	0 (0%)	20 (100%)
	C) Go Fish Game	16 (80%)	4 (20%)	0 (0%)	0 (0%)	20 (100%)
5	A) Bingo	19 (95%)	1 (5%)	0 (0%)	0 (0%)	20 (100%)
	B) Riddles	18 (90%)	2 (10%)	0 (0%)	0 (0%)	20 (100%)
	C) Phoneme Trail	20 (100%)	0 (0%)	0 (0%)	0 (0%)	20 (100%)
6	A) Dice	18 (90%)	2 (10%)	0 (0%)	0 (0%)	20 (100%)
	B) Syllabic Segmentation	17 (85%)	3 (15%)	0 (0%)	0 (0%)	20 (100%)

**Caption:** Activities: 2. Presentation and Articulatory Production of the Target Sound; 3. Target Sound Recognition and Auditory Discrimination with Minimal Pairs; 4. Minimal Pairs Strategy to Understand the Rule; 5. Practicing with words with the target sound in initial, medial, and final position; 6. Phonological Awareness

## DISCUSSION

The results indicated an agreement between EJs for the activities that address the PROCICLOS-A skills. Each activity corresponds to a skill, most of which originate from the cycles approach^(14)^, and all are equally important for overcoming phonological SSD. The auditory bombardment (activities 1 and 7) begins and ends each session to ensure that the child pays attention to the target sound, preparing attention for the target sound of the session. The auditory bombardment is especially interesting because several studies have shown that children with phonological SSD manifest difficulties in auditory perception, which can hinder the refinement of the phonological representation and production of the sound^(24)^.

Activity 3 also addresses auditory perception, encompassing the recognition and auditory discrimination of the target sound. The literature has highlighted the importance of auditory perception skills in phonological SSD intervention. A 2019 study found a relationship between types of speech errors and impaired auditory perception skills^(25)^. Another study, also from 2019, showed that all participants diagnosed with phonological SSD also had impaired auditory perception skills^(26)^.

Activity 2 of PROCICLOS-A presents and places the target sound using multimodal facilitating cues. It is considered of great importance, as it offers the child the first opportunity to produce the target sound of the session. Strategies use support cards with sketches of the articulators positioned to produce the sound, providing verbal guidance supported by visual and tactile biofeedback, often accompanied by ultrasound for sounds articulated with the tongue, followed by articulatory production practice. They form an important part of the intervention program, providing a complete presentation and initial practice of the target sound for the child. A systematic review on the use of speech ultrasound to work on various sounds suggested that this type of visual biofeedback facilitates the acquisition of sounds articulated by the tongue^(27)^.

Activity 4 uses minimal pairs to assist children in understanding and using the phonological rule involving the target sound and to eliminate the target phonological process. One advantage of using minimal pairs in an intervention approach is the use of homonyms to induce phonological learning in children. Two central aspects of intervention approaches in phonological SSD that employ minimal pairs stand out: pairing the target sound with its substitution/deletion in minimal pairs and intervention activities that create opportunities for practicing the word with the target sound in directed (word naming) and semi-directed (production of a sentence with the target word) situations through interactive games^(28)^. PROCICLOS-A strategies provide this situation and were considered appropriate by the EJs.

Activity 5 aims to work on the correct production of the target sound through word training with the sound in initial, medial, and final positions. The strategies provide several opportunities for the child to produce the target sound in directed situations (e.g., naming the target figure drawn in each round) and in free situations (e.g., developing sentences with the target word). The strategies allow for a high dose of target sound production training, ranging from 80 to 100. Several studies in the literature show that children with phonological SSD should have at least 100 opportunities to be exposed to and produce the target sound in target words during a session^(6,8,9)^.

The strategies of Activity 6, the last one worked on in the session, aim to develop phonological awareness skills by encouraging reflection on the phonological representation of the target sound. A systematic review shows that researchers and clinicians select intervention approaches and strategies to develop phonological awareness in children with speech and language disorders^(29)^. Not only does this skill predict reading and writing skills^(30)^, but more recent studies also show that improving phonological awareness skills has a positive impact on the phonological training of children with phonological SSD. Thus, using two phonological awareness strategies is an important complement to the work of adapting these children's speech.

The EJ agreement rate suggests evidence that the material developed for PROCICLOS-A meets its objectives, covering the various skills necessary for the improvement of children with SSD. The study on the program's implementation is already underway and will be published soon.

## CONCLUSION

The study demonstrated validation of the PROCICLOS-A activities and strategies, an intervention program for children with phonological SSD. The agreement rate among EJs was good for the proposed activities and strategies, demonstrating that they are appropriate for achieving their goals.

Thus, PROCICLOS-A contributes to clinical speech-language-hearing practice. Efficacy studies were conducted in parallel with this study to ensure the provision of an effective intervention for children with phonological SSD.
