# Supplementary material for: Content validation of the Adapted Cycles Intervention Program (PROCICLOS-A) for children with Speech Sound Disorder – Activities
Source: Codas. 2026 Feb 9;38(1):e20240378. doi: 10.1590/2317-1782/e20240378en (PMC12978331; doi:10.1590/2317-1782/e20240378en)
Supplement: Translation

# Translation: Validação de conteúdo do Programa de Intervenção do Ciclos Adaptado (PROCICLOS-A) para crianças com Transtorno dos Sons da Fala – Atividades

**DOI:** 10.1590/2317-1782/e20240378pt

**Authors:** Carolina Kuntz Ayub, Haydée Fiszbein Wertzner

**Affiliations:** 1 Programa de Pós-graduação em Ciências da Reabilitação, Departamento de Fonoaudiologia, Fisioterapia e Terapia Ocupacional, Faculdade de Medicina, Universidade de São Paulo – USP - São Paulo (SP), Brasil.; 2 Departamento de Fonoaudiologia, Fisioterapia e Terapia Ocupacional, Faculdade de Medicina, Universidade de São Paulo – USP - São Paulo (SP), Brasil.

**Keywords:** Fonoaudiologia, Fala, Transtorno Fonológico, Fonoterapia, Distúrbios da Fala

## Abstract

**Objetivo:**

Descrever e validar as atividades do Programa de Intervenção do Ciclos Adaptado (PROCICLOS-A) para crianças com transtorno dos sons da fala tipo fonológico idiopático.

**Método:**

Estudo prospectivo, transversal, de análise quantitativa. O PROCICLOS-A conta com 12 sessões, em que a cada seis sessões um processo fonológico é alvo da intervenção, sendo dois processos fonológicos e para cada um deles dois sons alvos são trabalhados, totalizando quatro sons alvos. Cada uma das 12 sessões conta com seis tipos de atividades: bombardeamento auditivo (no início e final da sessão); treino do ponto, modo articulatório e vozeamento do som alvo; reconhecimento auditivo do som alvo e discriminação auditiva com pares mínimos; atividades com pares mínimos para compreensão da regra; treino em palavras com o som alvo em posição inicial, medial e final; e atividades de consciência fonológica. Materiais específicos foram criados para executar cada estratégia. Participaram do estudo vinte juízes especialistas (JE), que fizeram o julgamento de todas as cinco atividades e suas 14 estratégias respectivas. Para as análises de concordância entre os juízes quanto as respostas referentes às atividades, foi aplicado um coeficiente alternativo, denominado AC1, proposto por Gwet (2014).

**Resultados:**

A concordância entre os JE para as atividades foi de 0,7125, considerado um valor de concordância moderado.

**Conclusão:**

As atividades do PROCICLOS-A para crianças com transtorno dos sons da fala apresentaram índice bom de concordância para todo o material produzido.

## INTRODUÇÃO

### Transtorno dos sons da fala

A aquisição fonológica da criança ocorre durante seu desenvolvimento, quando há um aumento do inventário fonético de seu sistema linguístico, mediado pela percepção auditiva, produção motora dos sons, bem como pelos aspectos cognitivo-linguísticos, que resultam na organização das regras fonológicas^(1)^.

Durante o desenvolvimento podem ser observadas omissões e/ou substituições de um ou mais sons da língua, denominados padrões de erros ou processos fonológicos, que são superados ao longo do tempo. Quando uma criança mantém os processos fonológicos para além da idade esperada, há indicação de uma alteração em seu sistema fonológico, e este quadro caracteriza-se como transtorno dos sons da fala (TSF), com grande ocorrência em crianças, principalmente pré-escolares. O TSF é um termo guarda-chuva, utilizado para se referir a qualquer combinação de dificuldades de percepção auditiva, produção motora e/ou representações fonológicas dos sons da fala, impactando diretamente na forma como o interlocutor fala^(2).^

O TSF mostra-se grande ocorrência em crianças em idade escolar e pré-escolar. Dentre os vários tipos, o TSF do tipo cognitivo-linguístico (ou fonológico) mostra-se como sendo o de maior incidência nesta faixa etária^(3)^. Muito se tem estudado a respeito das abordagens de intervenção que tem como foco o TSF do tipo fonológico idiopático^(4)^.

### Abordagens de intervenção

Na literatura, há várias abordagens de intervenção no TSF tipo fonológico idiopático (TSF – Fonológico). Dois aspectos importantes a serem considerados para a escolha de uma abordagem de intervenção são o fato de seus objetivos estarem claros, e seus elementos serem bem descritos, de modo a possibilitar sua aplicação, seja em âmbito clínico ou de pesquisa^(5)^. Um estudo de Hegarty et al.^(6)^, alerta que muitos fonoaudiólogos podem se sentir inseguros quanto à escolha da melhor abordagem de intervenção por não terem conhecimento a respeito de sua eficácia em relação ao que propõem tratar. Levando em consideração que uma intervenção busca reorganizar o sistema fonológico de crianças com TSF – Fonológico, as autoras observaram que a maioria dos profissionais opta por abordagens convencionais com foco em pares mínimos, produção motora e consciência fonológica.

Uma abordagem de intervenção bastante utilizada por fonoaudiólogos, denominada de ciclos^(7)^, tem como objetivo facilitar a aquisição de padrões fonológicos, por meio da seleção cuidadosa de fonemas em palavras que seriam utilizadas em atividades auditivas e cinestésicas, para aumentar as habilidades fonológicas da criança. Cada sessão da terapia englobava estratégias de bombardeamento auditivo e atividades variadas envolvendo palavras com o som alvo trabalhado. Eram fornecidas pistas táteis e auditivas durante a realização das atividades, visando o sucesso da correta produção do som, que iam diminuindo em sua frequência conforme a criança fosse mostrando melhor desempenho. A autora apresentou um modelo de intervenção, denominado abordagem de Ciclos, cujos objetivos de remediação eram facilitar a aquisição de padrões fonológicos, por meio da seleção cuidadosa de fonemas em palavras que seriam utilizadas em atividades auditivas e cinestésicas, para aumentar as habilidades fonológicas da criança.

### Validação de conteúdo de instrumento de intervenção

As pesquisas a respeito de abordagens de intervenção apontam a necessidade de se considerar a prática baseada em evidência^(8,9)^. Uma das questões importantes para a prática baseada em evidência são os resultados de pesquisa que indicam a eficácia de uma abordagem de intervenção. Um instrumento quer de avaliação ou de abordagem de intervenção com evidências da validade do conteúdo podem fornecer oportunidades suficientes para trabalhar as habilidades que se propõem^(10)^.

Baker et al.^(5)^, propuseram uma taxonomia para identificar elementos comuns e incomuns nas intervenções no TSF tipo fonológico, que tem como intuito a identificação de quais elementos estão descritos em uma abordagem de intervenção, bem com qual a relevância, impacto e objetivo de cada um desses elementos para a aplicação de uma determinada abordagem. Para tanto, selecionaram 15 abordagens de intervenção que foram analisadas quanto às suas estruturas, objetivos e aplicabilidades, de modo a oferecer transparência, nas descrições, tanto ao clínico como ao pesquisador. As autoras citam a importância da identificação clara de quais elementos estão descritos em uma abordagem de intervenção, bem com qual a relevância, impacto e objetivo de cada um desses elementos para a aplicação de uma determinada abordagem. Os elementos analisados podem variar, nas abordagens, quanto ao objetivo e foco da intervenção, objetivo das estratégias e das atividades escolhidas, sendo que estes podem influenciar a eficácia de cada abordagem de intervenção, assim como em seu uso em pesquisas e atendimentos clínicos. Destacam ainda que, no processo de construção de uma abordagem de intervenção, é necessário não somente comprovar sua eficácia, mas também ter a certeza de que cada parte proposta apresenta clareza no seu objetivo e que as atividades e estratégias atinjam o propósito a que se propõem. Um outro estudo^(9)^ aponta que a implementação de intervenções que possuem manual, treinamento e materiais adequados para sua aplicação, podem aumentar o uso de práticas baseadas em evidência entre os profissionais clínicos e pesquisadores.

Assim, uma etapa importante para observar quão eficaz e transparente é um programa de intervenção, é por meio de sua validação de conteúdo. Essa validação consiste em um julgamento de itens pré-selecionados, que são avaliados no grau em que cada elemento de um instrumento é relevante e representativo para o universo abordado em questão. É um processo que determina a precisão de determinados resultados, a partir de sua medição. Tem o objetivo de determinar se o instrumento em questão contempla todos os objetivos propostos, por meio de procedimentos psicométricos adequados^(11)^.

Quando se considera a validação de qualquer instrumento, dois aspectos devem ser levados em conta: a confiabilidade e a validade do que se está pesquisando. Para Phelan e Wren^(12)^, a confiabilidade é o grau em que uma ferramenta apresenta resultados estáveis ​​e consistentes. Entre seus subtipos, a ‘confiabilidade inter-avaliadores é uma medida de confiabilidade usada para avaliar o grau em que diferentes juízes (ou avaliadores) concordam em suas escolhas, sendo mais aconselhável para avaliar ilustrações, fotografias ou outro material não textual preparado para inclusão em uma publicação. Porém, segundo os mesmos autores, a confiabilidade, apesar de ser uma medida importante, não é suficiente. Os autores explicam, de maneira complementar, que a validação de conteúdo é utilizada para garantir o quanto o instrumento avaliado mede aquilo que se propõe a fazer, e quais itens estão adequados para aquele instrumento.

Alguns autores defendem ainda que a validação de conteúdo deve englobar três fases: identificação dos domínios, formação dos itens e construção do instrumento^(13)^. Também sugerem que a validação de conteúdo deve ser feita por meio da avaliação, por um comitê de juízes formado por, no mínimo, cinco especialistas, que devem receber instruções específicas sobre como avaliar cada item por meio do preenchimento de um questionário^(13)^.

Ao se verificar a validade de conteúdo das atividades e estratégias de uma abordagem de intervenção, pretende-se comprovar que tal abordagem tem a possibilidade de tratar de forma eficaz o que se propõe.

As hipóteses do presente estudo são: as atividades e suas respectivas estratégias propostas no PROCICLOS-A são adequadas para estimularem cada habilidade trabalhada. O presente estudo teve como objetivo descrever e validar as atividades do Programa de Intervenção do Ciclos Adaptado (PROCICLOS-A) para crianças com transtorno dos sons da fala.

## MÉTODO

Este é um estudo prospectivo, transversal, de análise quantitativa, sendo os materiais apresentados relativos à uma abordagem de intervenção específica, o Programa de Intervenção do Ciclos Adaptado (PROCICLOS-A). O estudo foi aprovado pela Comissão de Ética CAAE 87068318.2.0000.0065, número 6.500.529. O termo de Consentimento foi elaborado em uma versão virtual, e anexado junto ao formulário que foi enviado aos Juízes Especialistas (JE), para ser preenchido antes do julgamento das atividades e estratégias.

### PROCICLOS-A

O PROCICLOS-A é um programa de intervenção fundamentado na abordagem dos ciclos de Hodson e Paden^(14)^, com foco na interação dos processos cognitivo-linguístico, perceptivo e de produção motora da fala. O objetivo central deste tipo de abordagem de intervenção, que permeia os processos de aquisição e domínio dos sons da língua e suas regras fonológicas, é o fato de ser um processo gradual. Isso significa que novos sons são introduzidos para serem trabalhados, mesmo que os anteriores ainda não tenham sido plenamente aprendidos. A intervenção ocorre em formato de ciclos: a cada duas sessões, muda-se o som alvo, sem critérios de acertos necessários para que esta mudança ocorra. Ainda, este tipo de abordagem de intervenção conta com atividades do tipo bombardeamento auditivo, treino articulatório e consciência fonológica^(14-18)^.

O Programa de Intervenção do Ciclos Adaptado (PROCICLOS-A) é uma revisão da proposta de intervenção de intervenção do Abordagem de Ciclos Adaptado^(19)^, que por sua vez é uma adaptação da abordagem dos ciclos proposta por Hodson e Paden^(14)^.

O PROCICLOS-A foi elaborado em laboratório de pesquisa, para ser aplicado em crianças com TSF idiopático tipo fonológico. A proposta do programa é eliminar a ininteligibilidade de fala por meio de atividades que estimulem a percepção auditiva dos sons, forneça pista necessárias para a produção adequada dos sons da fala utilizando as regras fonológicas da língua e esperadas para a idade da criança, ou seja, uma abordagem integrada, como sugerido por Wren et al.^(4)^.

Assim como na abordagem dos ciclos, no PROCICLOS-A é adotada a estratégia cíclica. No PROCICLOS-A, são selecionados dois processos fonológicos e dois sons alvos em cada um deles para serem trabalhados durante as 12 sessões, totalizando quatro sons alvos (Figura 1). Inicia-se pelo processo de maior ocorrência e, que, portanto, esteja comprometendo com maior intensidade a inteligibilidade de fala. Também, em geral, seleciona-se aqueles processos fonológicos que são eliminados mais cedo durante o desenvolvimento. Em relação ao som alvo, elege-se para o início aqueles que são estimuláveis. Um destaque é que no PROCICLOS-A, para trabalhar a eliminação dos processos fonológicos, optou-se pela utilização de pares mínimos com oposição mínima, em que há apenas a diferença em um fonema, e por apenas um traço contrastivo, como por exemplo em /vaka/ x /faka/.

**Figura 1:** Fluxograma da distribuição dos processos fonológicos e sons alvos no PROCICLOS-A

Cada uma das 12 sessões conta com seis tipos de atividades que promovem diferentes habilidades: bombardeamento auditivo (no início e final da sessão); colocação do som alvo; reconhecimento auditivo do som alvo e discriminação auditiva com pares mínimos; atividades com pares mínimos com oposição mínima para compreensão da regra; treino em palavras com o som alvo em posição inicial, medial e final; e atividades de consciência fonológica. No Quadro 1, observa-se o objetivo de cada atividade realizada nas 12 sessões do PROCICLOS-A, bem como as estratégias aplicadas.

**Quadro 1:** Objetivo das atividades e respectivas estratégias do PROCICLOS-A

Atividade	Objetivo	Estratégia
Atividade 1: “Bombardeamento Auditivo	A criança deve ouvir atentamente às palavras com o som alvo.	Leitura de palavras dissílabas que iniciam com os sons-alvo do processo fonológico que está sendo trabalhado.
Atividade 2: "Apresentação e Produção Articulatória do Som Alvo".	Auxiliar a criança a produzir os sons, por meio de pistas facilitadoras multimodais, ou seja, pistas auditivas, visuais e táteis.	2.1. Apresentação do som alvo – A) Cartas de produção guiada dos sons da fala.
		2.1. Apresentação do som alvo – B) Ultrassonografia de fala.
		2.2. Prática da produção articulatória do som alvo.
Atividade 3: "Reconhecimento do Som Alvo e Discriminação Auditiva com Pares Mínimos".	Auxiliar a criança a reconhecer e discriminar o som alvo na palavra.	3.1. Reconhecimento Auditivo do som alvo – A) Jogo Pula-Pula
		3.1. Reconhecimento Auditivo do som alvo – B) Jogo do Tapa Certo
		3.2 Discriminação Auditiva do som alvo – Jogo do Agrupamento
Atividade 4: "Estratégia com Pares Mínimos para Compreensão da Regra".	Auxiliar a criança na compreensão e utilização da regra fonológica.	4. A) Jogo da Memória
		4. B) Jogo do Dominó
		4. C) Jogo do Mico
Atividade 5: "Treino em palavras com o som alvo em posição inicial, medial e final".	Trabalhar a correta produção do som alvo em posição inicial, medial e final; contribuir para estimular a memória fonológica de trabalho e auxilia no reconhecimento dos sons trabalhados.	5. A) Bingo
		5. B) O que é O que é
		5. C) Trilha de Fonemas
Atividade 6:"Consciência Fonológica".	Refletir a respeito do som alvo e sua representação fonológica.	6. A) Dado
		6. B) Segmentação Silábica

### Sujeitos

Vinte Juízes Especialistas (JE) fonoaudiólogos, participaram do processo de validação do conteúdo do programa de intervenção PROCICLOS-A. Os critérios de inclusão para os JE foram: serem fonoaudiólogos, com mestrado completo ou doutorado (cursando ou já finalizado), com experiência de atuação na área de TSF – Fonológico. Os convites aos JE ocorreram por meio do aplicativo de mensagens instantâneas *WhatsApp* no celular ou por e-mail. No e-mail enviado aos JE, inicialmente, a pesquisadora se apresentou e em seguida expôs brevemente o estudo, finalizando a mensagem com o convite para participar como JE, e os links de cada formulário que deveria ser respondido. Todos os JE que aceitaram participar, assinaram um Termo de Consentimento Livre e Esclarecido, que foi apresentado para cada um dos JE responderem, no início de cada formulário, que contou com a seguinte pergunta: “Você concorda em participar dessa pesquisa?”, seguido de duas opções para assinalar: “Concordo” ou “Não Concordo”.

### Procedimentos

Para a análise das atividades e estratégias foi preparado formulário específico, que foi enviado por um *link*, para os JE. Concluída a fase de preenchimento de todos os formulários por cada um dos JE, as respostas foram organizadas em tabela no programa *Excel*.

### Atividades

O formulário respondido por cada um dos 20 JE continha 14 perguntas relativas às estratégias de cada uma das atividades.

O formulário contava com uma breve explicação de cada uma das atividades, e qual ou quais habilidades cada atividade pretendia estimular, ou seja, o objetivo que se pretendia atingir com a atividade. Em seguida, eram apresentadas as estratégias elaboradas para atingir os objetivos definidos pela respectiva atividade. O JE precisou analisar e decidir se as estratégias eram adequadas para atingir os objetivos propostos em cada atividade. Essa análise foi feita utilizando a Escala *Likert*: “Concordo Totalmente”, “Concordo Parcialmente”, “Discordo Parcialmente”, “Discordo Totalmente”, “Não se Aplica”. Um exemplo deste formulário encontra-se demonstrado na Figura 2.

**Figura 2:** Exemplo de Formulário para “Atividades”

### Análise estatística

A concordância entre os JE foi calculada por meio da estatística Kappa de Fleiss, que é uma extensão da estatística Kappa de Cohen para mais de dois juízes. Porém, segundo os autores Gama^(20)^, Santos^(21)^ e Wongpakaran et al.^(22)^, os coeficientes Kappa de Cohen e de Fleiss apresentam desempenhos deficientes em determinadas situações, como o caso deste estudo, em que a proporção de ocorrência de uma categoria de resposta é muita alta quando comparada às demais, resultando em valores dos coeficientes baixos, apesar de a soma das proporções em que os JE concordaram ser alta. Para adequar as análises, Gama^(20)^, Santos^(21)^ e Wongpakaran et al.^(22)^ recomendam o uso de um coeficiente alternativo, denominado AC1, proposto por Gwet^(23)^, aplicado neste estudo. A classificação da concordância, segundo o valor do coeficiente AC1, é apresentada na Tabela 1.

**Tabela 1:** Avaliação da concordância segundo o valor do coeficiente AC1

Valor do coeficiente	Concordância
< 0,20	Desprezível
0,21 a 0,40	Mínima
0,41 a 0,60	Moderada
0,61 a 0,80	Boa
0,81 a 1,00	Excelente

## RESULTADOS

Foram obtidas as respostas dos 20 JE para cada um dos formulários enviados. Todos os JE responderam ao formulário “Atividades”. A concordância entre os JE é apresentada considerando cada um dos itens analisados.

### Descrição da concordância dos JE para as atividades

A análise da concordância entre os 20 JE para as atividades do PROCICLOS-A, com suas respectivas estratégias, indicou o valor de AC1 0,7125, considerado um valor de concordância moderado. Para verificar a influência de cada atividade no valor do coeficiente AC1, esse coeficiente foi recalculado excluindo uma atividade por vez. Se o valor de AC1 obtido, excluindo uma atividade, fosse inferior ao obtido originalmente, com todas as atividades incluídas (AC1 0,7125), o resultado indicaria que a atividade excluída contribui para uma melhor concordância geral. Já, se o valor for superior a 0,7125, indica que a atividade excluída piora a concordância geral entre os juízes quando mantida. A Tabela 2 mostra que as atividades 2.1 – A), 2.2, 3.1 – A), 3.1 – B), 4 – A), 4 – B), 4 – C) e 6.2 – B), apesar de causarem uma leve piora no índice de concordância geral, o valor de AC1 obtido ainda se mantém como concordância moderada, indicando que as atividades se mostram adequadas e, portanto, podem ser mantidas.

**Tabela 2:** Valor do coeficiente AC1 após a exclusão de cada atividade

Atividades	AC1
2.1 – A)	**0,7193**
2.1 – B)	0,6996
2.2	**0,7292**
3.1 – A)	**0,7247**
3.1 – B)	**0,7193**
3.2 – A)	0,6912
4 – A)	**0,7129**
4 – B)	**0,7193**
4 – C)	**0,7193**
5 – A)	0,6976
5 – B)	0,7057
5 – C)	0,6885
6 – A)	0,7057
6 – B)	**0,7129**

A análise da proporção de ocorrência das alternativas de respostas dos JE indica que, para todas as atividades, a resposta “Concordo Totalmente” variou entre 70% e 100%, sendo que somente para dois itens houve um “Discordo Parcialmente” e uma resposta de “Não se aplica”, para as atividades 2.1 – B) e 3.2 – A), respectivamente (Tabela 3).

**Tabela 3:** – Proporção de ocorrência das respostas dos juízes especialistas

Atividades		Concordo totalmente	Concordo parcialmente	Discordo parcialmente	Não se aplica	Total
2.1	Apresentação do som alvo – A)	16 (80%)	4 (20%)	0 (0%)	0 (0%)	20 (100%)
	Apresentação do som alvo – B)	17 (85%)	2 (10%)	1 (5%)	0 (0%)	20 (100%)
2.2	Prática da Produção Articulatória do Som alvo	14 (70%)	6 (30%)	0 (0%)	0 (0%)	20 (100%)
3.1	A) Jogo Pula-Pula	15 (75%)	5 (25%)	0 (0%)	0 (0%)	20 (100%)
	B) Jogo do Tapa Certo	16 (80%)	4 (20%)	0 (0%)	0 (0%)	20 (100%)
3.2	A) Jogo do Agrupamento	18 (90%)	1 (5%)	0 (0%)	1 (5%)	20 (100%)
4	A) Jogo da Memória	17 (85%)	3 (15%)	0 (0%)	0 (0%)	20 (100%)
	B) Jogo do Dominó	16 (80%)	4 (20%)	0 (0%)	0 (0%)	20 (100%)
	C) Jogo do Mico	16 (80%)	4 (20%)	0 (0%)	0 (0%)	20 (100%)
5	A) Bingo	19 (95%)	1 (5%)	0 (0%)	0 (0%)	20 (100%)
	B) O que é, O que é	18 (90%)	2 (10%)	0 (0%)	0 (0%)	20 (100%)
	C) Trilha de Fonemas	20 (100%)	0 (0%)	0 (0%)	0 (0%)	20 (100%)
6	A) Dado	18 (90%)	2 (10%)	0 (0%)	0 (0%)	20 (100%)
	B) Segmentação Silábica	17 (85%)	3 (15%)	0 (0%)	0 (0%)	20 (100%)

**Legenda:** Atividades: 2. Apresentação e Produção Articulatória do Som alvo; 3. Reconhecimento do Som alvo e Discriminação Auditiva com Pares Mínimos; 4. Estratégia com Pares Mínimos para Compreensão da Regra; 5. Treino em palavras com o som alvo em posição inicial, medial e final; 6. Consciência Fonológica

## DISCUSSÃO

Os resultados indicaram que houve concordância entre os JE para as atividades propostas para trabalhar as habilidades do PROCICLOS-A. Cada uma das atividades propostas corresponde a uma habilidade, sendo que a maior parte é originária da abordagem dos ciclos^(14)^, e todas têm igual importância para a superação do TSF – Fonológico. O bombardeamento auditivo (atividade 1 e 7) que inicia e finaliza cada sessão, tem como objetivo que a criança fique atenta ao som alvo, preparando a atenção ao som alvo da sessão. O bombardeamento auditivo é especialmente interessante porque vários estudos têm mostrado que crianças com TSF – Fonológico manifestam dificuldades de percepção auditiva, as quais podem dificultar o refinamento da produção e representação fonológica do som^(24)^.

Na atividade 3 também se aborda a percepção auditiva englobando o reconhecimento e discriminação auditiva do som alvo. A importância da habilidade de percepção auditiva na intervenção do TSF – Fonológico tem sido destacada na literatura. Um estudo de 2019 constatou a relação entre tipos de erros na fala e habilidades de percepção auditiva alteradas^(25)^. Um outro estudo, também de 2019, mostrou que todos os participantes com diagnóstico de TSF – Fonológico também apresentavam alterações nas habilidades de percepção auditiva^(26)^.

A Atividade 2 do PROCICLOS-A, na qual ocorre a apresentação e colocação do som alvo, por meio de pistas facilitadoras multimodais, é considerada de grande importância, na medida em que oferece à criança as primeiras oportunidades de produção do som alvo da sessão. Por fornecer orientações verbais, com apoio de *biofeedback* visual e tátil, as estratégias com uso de cartas de apoio com esboço dos articuladores na posição de produção, acompanhado muitas vezes da ultrassonografia para os sons que têm a língua como um dos articuladores, seguido de prática da produção articulatória, formam uma parte importante do programa de intervenção, proporcionando uma apresentação e prática inicial do som alvo completa para a criança. Em uma revisão sistemática a respeito do uso de ultrassonografia de fala para trabalhar vários sons, sugeriu que o esse tipo de *biofeedback* visual facilita na aquisição de sons cujo articulador é a língua^(27)^.

Para auxiliar a criança na compreensão e utilização da regra fonológica que envolve o som alvo, na Atividade 4 utilizam-se estratégias com pares mínimos que visam eliminar o processo fonológico alvo. Uma das vantagens de usar pares mínimos em uma abordagem de intervenção é o de fazer uso de homônimos para induzir um aprendizado fonológico na criança. São apontados dois aspectos centrais para abordagens de intervenção no TSF – Fonológico que empregam par mínimo: pareamento do som alvo com sua substituição/omissão em pares mínimos, e atividades de intervenção que criem oportunidades para treino da palavra com o som alvo em situação direcionada (nomeação da palavra) e semi-direcionada (produção de uma frase com a palavra alvo), por meio de jogos interativos^(28)^. As estratégias propostas no PROCICLOS-A proporcionam essa situação e foram consideradas adequadas pelos JE.

Na Atividade 5, que visa trabalhar a produção correta do som alvo, por meio do treino em palavras, com o som em posição inicial, medial e final, as estratégias proporcionam diversas oportunidades de a criança produzir o som alvo, em situações direcionadas, como na nomeação da figura alvo sorteada a cada rodada e em situações livres, como nos momentos em que elaboram frases com a palavra alvo. As estratégias possibilitam uma alta dose do treino de produção do som alvo, que varia entre 80 e 100. Na literatura, várias pesquisas mostram que deve haver pelo menos 100 oportunidades da criança com TSF – Fonológico ser exposta e/ou produzir o som alvo em palavras alvo, durante uma sessão^(6,8,9)^.

Para a última atividade trabalhada em sessão, as estratégias da Atividade 6 visam trabalhar a habilidade de consciência fonológica, por meio de estratégias que proporcionam a reflexão da representação fonológica do som alvo. Uma revisão sistemática mostra que pesquisadores e clínicos selecionam abordagens de intervenção e estratégias para trabalhar a consciência fonológica de crianças com alterações de fala e linguagem^(29)^. Não somente esta habilidade é preditora das habilidades de leitura e escrita^(30)^, estudos mais recentes mostram que melhorar as habilidades de consciência fonológica tem um impacto positivo no treino fonológico de crianças com TSF – Fonológico. Dessa forma, a presença de duas estratégias de consciência fonológica são um complemento importante no trabalho de adequação da fala dessas crianças.

O índice de concordância dos JE sugere evidências que o material elaborado para o PROCICLOS-A cumpre com seus objetivos, cobrindo as várias habilidades necessárias para promover a melhora das crianças com TSF. O estudo referente à aplicação do programa já está em andamento e será publicado em breve.

## CONCLUSÃO

O estudo realizado mostrou evidências de validação das atividades e suas respectivas estratégias do PROCICLOS-A, programa de intervenção para crianças com TSF – Fonológico. O índice de concordância entre os JE foi bom para as atividades e estratégias propostas, evidenciando que estas estão adequadas para promoverem o que foi proposto para cada uma delas.

Desta forma, o PROCICLOS-A traz contribuições para a prática clínica fonoaudiológica. Estudos de eficácia foram desenvolvidos em paralelo ao presente estudo com o intuito de se certificar do oferecimento de uma intervenção eficaz para crianças com TSF – Fonológico.
